# Phytochemical analysis and in vitro anthelmintic activity of *Lophira lanceolata* (Ochnaceae) on the bovine parasite *Onchocerca ochengi* and on drug resistant strains of the free-living nematode *Caenorhabditis elegans*

**DOI:** 10.1186/s12906-017-1904-z

**Published:** 2017-08-14

**Authors:** Justin Kalmobé, Dieudonné Ndjonka, Djafsia Boursou, Jacqueline Dikti Vildina, Eva Liebau

**Affiliations:** 1 Department of Biological Sciences, Faculty of Science, University of Ngaoundéré, PO Box 454, Ngaoundéré, Cameroon; 2 Department of Molecular Physiology, Institute for Zoophysiology, Schlossplatz 8, 48143 Muenster, Germany

**Keywords:** *Onchocerca ochengi*, Anthelmintic, *Lophira lanceolata*, Drug resistant strains, Acute toxicity, Traditional healers

## Abstract

**Background:**

Onchocerciasis is one of the tropical neglected diseases (NTDs) caused by the nematode *Onchocerca volvulus*. Control strategies currently in use rely on mass administration of ivermectin, which has marked activity against microfilariae. Furthermore, the development of resistance to ivermectin was observed. Since vaccine and safe macrofilaricidal treatment against onchocerciasis are still lacking, there is an urgent need to discover novel drugs. This study was undertaken to investigate the anthelmintic activity of *Lophira lanceolata* on the cattle parasite *Onchocerca ochengi* and the anthelmintic drug resistant strains of the free living nematode *Caenorhabditis elegans* and to determine the phytochemical profiles of the extracts and fractions of the plants.

**Methods:**

Plant was extracted in ethanol or methanol-methylene chloride. *O. ochengi*, *C. elegans* wild-type and *C. elegans* drug resistant strains were cultured in RPMI-1640 and NGM-agar respectively. Drugs diluted in dimethylsulphoxide/RPMI or M9-Buffer were added in assays and monitored at 48 h and 72 h. Worm viability was determined by using the MTT/formazan colorimetric method. Polyphenol, tannin and flavonoid contents were determined by dosage of gallic acid and rutin. Acute oral toxicity was evaluated using Swiss albino mice.

**Results:**

Ethanolic and methanolic-methylene chloride extracts killed *O. ochengi* with LC_50_ values of 9.76, 8.05, 6.39 μg/mL and 9.45, 7.95, 6.39 μg/mL respectively for leaves, trunk bark and root bark after 72 h. The lowest concentrations required to kill 50% of the wild-type of *C. elegans* were 1200 and 1890 μg/mL with ethanolic crude extract, 1000 and 2030 μg/mL with MeOH-CH_2_Cl_2_ for root bark and trunk bark of *L. lanceolata*, respectively after 72 h. Leave extracts of *L. lanceolata* are lethal to albendazole and ivermectin resistant strains of *C. elegans* after 72 h. Methanol/methylene chloride extracted more metabolites. Additionally, extracts could be considered relatively safe.

**Conclusion:**

Ethanolic and methanolic-methylene chloride crude extracts and fractions of *L. lanceolata* showed in vitro anthelmintic activity. The extracts and fractions contained polyphenols, tannins, flavonoids and saponins. The mechanism of action of this plant could be different from that of albendazole and ivermectin. These results confirm the use of *L. lanceolata* by traditional healers for the treatment of worm infections.

**Electronic supplementary material:**

The online version of this article (doi:10.1186/s12906-017-1904-z) contains supplementary material, which is available to authorized users.

## Background

Neglected Tropical Diseases (NTDs) remain major public health problems and the most important obstacles to development of sub-saharian Africa [1]. Despite renewed interest in the prevention and control of those diseases, lymphatic filariasis (LF) and onchocerciasis continue to spread in the developing countries causing disabilities [2]. Onchocerciasis is a filarial disease caused by *Onchocerca volvulus* and transmitted by the blackflies of the genus *Simulium* [3]. The pathology of the disease is characterized by cutaneous manifestations such as nodules, dermatitis and ultimately ocular syndrome. Globally, within the 37 million of infected people, 99% live in Africa with 500,000 visually impaired and 270,000 blind [4]. In the Adamawa region of Cameroon, the prevalence of human and animal onchocerciasis has been estimated at 30% and 65% respectively [5]. Onchocerciasis causes disability, social stigmatization and forces the affected populations to abandon the endemic areas, which usually have high agricultural potential [5]. Thus, a high burden of onchocerciasis in a country leads primarily to low productivity and consequently to an economical loss and a slowdown of development [6]. Several approaches were attempted to control onchocerciasis in Human. The control started with vector control involving spraying of insecticides and larvicides [7] followed by mass treatment using various combinations of drugs including ivermectin which is actually the recommended molecule against onchocerciasis [8]. Although this drug reduces significantly transmission of the disease, its filaricidal effect is limited only to the juvenile form of the parasite [9]. Numerous studies have revealed lethal adverse effects on patients co-infected with Onchocerciasis and loasis that ranked from fatigue to consciousness disorders and death [10]. In some Asian and African countries, 80% of the population depends on traditional medicine for primary health care [11]. The herbal medicines are therefore the most lucrative form of traditional medicine, generating billions of dollars in revenue [11]. Based on current knowledge of the plants, their use in traditional treatment of parasitic diseases and their multiple beneficial properties for humans, there is an opened possibility for new anthelmintic from medicinal plants. Traditional healers in Cameroon use *Lophira lanceolata* for the treatment of human onchocerciasis. *L. lanceolata* is been used in traditional medicine against constipation, diarrhoea, dysentery, menstrual pain (women) as concoction and infusion of bark of the roots and trunk [12]. The pharmacological activity studies of this plant revealed that it possesses antipyretic activity, cure potential on chronic wound, antimicrobial activities against some fungi and bacteria [13], antidiarrhoeal and anti-plasmodial effects [14]. However anthelmintic activity of this plant has not yet been evaluated on filarial worms. In this study, we investigated the claimed filaricidal activities of *L. lanceolata* against the bovine parasite *Onchocerca ochengi.* This parasite is considered as an appropriate model to study anthelmintic activities. *C. elegans* serves also as a suitable model organism for research on nematode parasites is used as well [15]. Extracts of several African plant species have shown activity against parasitic nematodes and the free-living nematode *C. elegans* [16]. The present study investigates the in vitro antifilarial activity of both crude extracts and chromatographic fractions of extracts of *L. lanceolata* leaves, trunk bark and root bark against *O. ochengi* adult forms, *C. elegans* wild type as well as drug resistant strains. Additionally we investigated the acute toxicity and the phytochemical profiles of the extracts and fractions of the plants.

## Methods

### Plant material and chemicals

Leaves, trunk barkand root bark of *Lophira lanceolata* (Ochnaceae) were collected in Ngaoundere, Adamawa region of Cameroon and identified by Dr. Tchobsala of Department of Biological Sciences, University of Ngaoundere (Cameroon). Voucher specimens have been registered under Number 3512/SRFK-CAM at the National Herbarium in Yaounde (Cameroon). All chemicals were purchased from Sigma (Deisenhofen, Germany).

### Preparation of extracts and fractionation

Plant extracts were prepared according to the method described by Ndjonka et al. [17] and Abdullahi et al. [18]. Briefly, 50 g of powdered plant organs were extracted in 500 mL of ethanol-distilled water (70:30) and MeOH-CH_2_Cl_2_ (50:50 *v*/v) for 48 h at room temperature, centrifuged (3500×g, 10 min) and filtered over filter papers No. 413 (VWR International, Darmstadt, Germany). The clear filtrate was concentrated by a rotatory evaporator at 40 °C under reduced pressure, and lyophilized. The resulting powder was stored at 4 °C for further investigation. For fractionation, dried powder of leaves (1.5 kg) and root bark (2 kg) were macerated with 4 L of MeOH-CH_2_Cl_2_ (50:50 *v*/v) for 48 h then filtered with wattman paper No. 1 [18]. The organic solvents were concentrated under reduced pressure at 40 °C, using rotary evaporator (Buchi Rotavapor R-210, Germany) to yield crude extracts of leaves (5.24%) and root bark (3.94%) [19]. Each crude extract (78.61 g of leaves and 78.87 g of root barks’) was re-suspended in MeOH-CH_2_Cl_2_ then partitioned with hexane (FH) (1:0 *v*/v), hexane: acetate (FHAE) (8:2 *v*/v), hexane: acetate (FHAEt) (6:4 *v*/v), acetate (FAE) (1:0 *v*/v), acetate: methanol (FAEM) (8:2 *v*/v), acetate: methanol (FAEMe) (7:3 *v*/v) and methanol (FM) (1:0 *v*/v) successively [18]. The partitions were concentrated under reduced pressure to dryness and stored at 4 °C. Small amount were then submitted to bioassay and phytochemical analysis. The dried plant extracts and partitions were diluted with 0.2% dimethylsulphoxide (DMSO) in M9-buffer (1.5 g KH2 PO4, 3 g Na2 HPO4, 2.5 g NaCl, 0.5 mL 1 M MgSO4) for *C. elegans* or RPMI-1640 for *O. ochengi* to a final concentration of 100 mg/mL. The solution was mixed thoroughly and stored for anthelminthic activity determination against *O. ochengi* and *C. elegans*.

### Isolation and culture of *O. ochengi* and *C. elegans*

The isolation of *O. ochengi* adult worms was done following the method used by Ndjonka et al. [17]. Briefly, pieces of infected umbilical skin bought from the slaughterhouse at Ngaoundere were brought to the laboratory for the removal of nodules and their dissection. Dissection was carried out under dissecting microscope (maximum magnification ×50). Adult worms were isolated and washed following standard procedures. Their viability was ascertained. Viable worms were then collected and numbered for anthelmintic assays according the method of Borsboom et al. [20].

The following *C. elegans* strains were used: N2 Bristol, referred to as wild type (WT); levamisole-resistant strains CB211 (*lev-1*(e211) IV), the albendazole-resistant strain CB3474 (*ben-1*(e1880) III) and ivermectin-resistant strains VC722 (*glc-2*(ok1047) I). All strains were obtained from the *Caenorhabditis* Genetic Centre (CGC, Minneapolis, MN, USA). *C. elegans* culture was performed on a solid medium NGM (Nematode Growth Medium) - agar as well as in M9 liquid medium. The solid culture medium NGM-Agar was made by dissolving in 1000 mL of distilled water 17 g of agar, 3 g of NaCl and 2.5 g peptone from casein, and then autoclaved. 25 mL of 1 M KH_2_PO_4_ / K_2_HPO_4_; 1 mL of 1 M MgSO_4_; 1 mL of 1 M CaCl_2_; 1 mL cholesterol were added prior to use. This culture was carried out in Petri dishes. On the medium was added a lawn of *Escherichia coli* OP50 solution and 0.5 μL of M9 containing *C. elegans* larvae. The Petri-dish was observed under a microscope to check worm’s viability then sealed with a film paper. Those dishes were then incubated at 20 °C until obtention of gravid worms prior to the synchronization [17].

### Anthelmintic screening assay

Following the protocol Borsboom et al. [20], six adults of *O. ochengi* were incubated with increasing concentrations (0 to 40 μg/mL) of plant extracts in RPMI supplemented with 100 UI/mL/100 μg/mL of penicillin/streptomycin. Positive controls are ivermectin, albendazole and levamisole. The tubes were incubated at 37 °C and the mortality was checked by using the MTT/formazan assay after 48 h or 72 h [17].

After chlorox treatment [17], isolated eggs of *C. elegans* were poured on NGM-agar plates to initiate synchronous culture. After eggs-hatching, the synchronized L4/young adults were transferred from solid medium into 24-well sterile plates containing M9-buffer (each well contains 10 young worms). To *C. elegans* cultures, increasing concentrations (0 – 8 × 10^3^ μg/mL) of leaves, trunk bark and root bark extracts of *L. lanceolata* were added. Worm mortality rate was determined after 48 h or 72 h at 20 °C. Positive controls (ivermectin, levamisole and albendazole) were assessed using the same method (0**–**20 μg/mL). 0.2% DMSO was used as negative control. Each experiment was conducted in three independent duplicates.

### Worm mortality and LC_50_ determination

The death was assessed by the MTT/formazan assay. The worms were placed in a well of a 96-well plate containing 200 μl of 0.5 mg/mL MTT in PBS and incubate under the culture condition for 30 min. LC_50_ values were determined by calculation using Log/probit method [21].

### Phytochemical test

The tannins content was determined as follows: 200 μL of the sample were mixed with 35% (*w*/*v*) Na_2_CO_3_ and 100 μL of Folin-Ciocalteu (FC) reagent. The solution was vortexed one minute, incubated five minutes and the absorbance at 640 nm was then measured. The results were expressed in mg equivalent of gallic acid per gram of dry materials (mg of GAE/g) [22].

The quantification of polyphenols was carried out using the method of Folin-Ciocalteu which consists in an evaluation of gallic acid amount in a serie of dilution of its aqueous solution [23]. A titration curve of gallic acid at 765 nm was performed. Briefly 50 μL of the sample was mixed with 200 μL of 35% (*w*/*v*) Na_2_CO_3_ and 250 μL of 1/10 (*v*/v) FC reagent. The mixture were agitated and incubated in darkness at 40 °C for 30 min and the absorbance was read at 765 nm using a spectrophotometer (UV-biowave Cambridge, England). The results were expressed in mg equivalent of gallic acid per grams of dry materials (mg of GAE/g). Polyphenols quantity was determined by calculation from the standard curve of gallic acid titration.

The determination of flavonoids content was performed according to the method described by Wolfe et al. [23]. To 0.1 g of each extract, 2 mL of extraction solvent (140:50:10 methanol-distilled water-acetic acid) was added to the plant extract. The mixture was filtered using a wattman paper and extraction’s solvent was added. Two hundred and fifty μL of the solution was transferred to a 14 mL tube and top up to 5 mL using distilled water. The obtained solution was the analysis solution. For titration, to 1 mL of analysis solution, 200 μL of distilled water and 500 μL of aluminum chlorite solution (133 mg of AlCl_3_ and 400 mg sodium acetate in 100 mL distilled water) were then added, and the solution mixed by vortexing. The absorbance was read at 430 nm. A standard titration curve was made using rutin. The amount of flavonoids was expressed as mg of rutin/g of dry materials.

### Acute toxicity studies of active methanolic/methylene chloride extract of *Lophira lanceolata* in *Swiss* albino mice

Mice were purchased from LANAVET and kept in a room temperature at 22 ± 2 °C with a relative humidity of 55 ± 1 °C. They were kept in cages one week for acclimatization, feed with standard rodent food before testing. The acute oral toxicity was realized according to the recommendations and guidelines of the Organization of Cooperation and Economic Development (OECD) [24] for chemicals’ tests. The animal experience was authorized by the regional delegate of livestock; fisheries and animal industries (N° 075/16/L/RA/DREPIA).

Ethanolic and methanolic/methylene chloride extracts of leaves and barks of *Lophira lanceolata* suspended in water were administered in a single oral dose to *Swiss* albino mice (22.02 to 30.1 g). Six females and six males were used for each dose. They were deprived of food but not water 4 h prior to the administration of the test substance. The doses of 1500; 3000 and 5000 mg/Kg of body weight were orally administered using a feeding needle. The control group received an equal volume of water as vehicle. Observation of toxic symptoms was made and recorded systematically after 1, 2, 4 and 6 h post administration. Finally, the number of survivors was recorded after 24 h and these animals were then maintained for further 14 days with daily observation [25].

### Data analysis

LC_50_ values were calculated using Log-probit method with SPSS 16.0 software. Data were expressed as mean ± standard error on the mean (M ± SEM). Data comparison was done using analysis of variances (one way **-** ANOVA) followed by multiple tests of comparison of Bonferroni. The calculation of the phytochemical metabolites of the plant was performed using standard curve formula y = ax + b, where y is the absorbance and x is the content in mg for g of dry materials. The curves and graphs were plotted using Graph Pad prism 5.10. Values of *P* < 0.05 were considered statistically significant.

## Results

### Anthelmintic activity of ethanolic and methanolic/methylene chloride extracts of *L. lanceolata* on *O. ochengi*

**Fig. 1:**
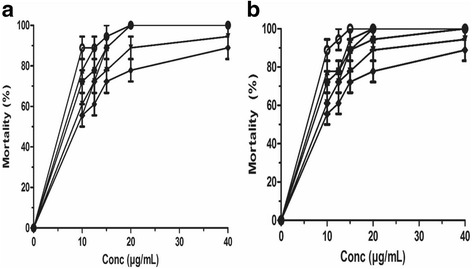
Activity against *O. ochengi* with (**a**) crude ethanolic and (**b**) MeOH-CH_2_Cl_2_ extracts from *L. lanceolata* (●) leaves, (■) trunk bark, (▲) root bark; (▼) Levamisole, (♦) Albendazole and (○) Ivermectin 72 h post-exposure. Data are mean ± SEM from three independent duplicate experiments

The anthelmintic activities of leaves, trunk bark and root bark of *L. lanceolata* on *O. ochengi* adult and on *C. elegans* WT were evaluated in terms of mortality after 48 h and 72 h of incubation. Ethanolic and MeOH-CH_2_Cl_2_ extracts of leaves, trunk bark and root bark of *L. lanceolata* killed *O. ochengi* completely with LC_100_ = 20 μg/mL after 72 h incubation (Fig. 1a and b). Their LC_50_ values were consigned in Table 1. Leaves, trunk bark and root bark killed worms with LC_50_ of 9.76 ± 0.49 μg/mL, 8.05 ± 1.15 μg/mL, 6.39 ± 2.11 μg/mL and 9.45 ± 0.37 μg/mL, 7.95 ± 1.70 μg/mL, 6.39 ± 2.11 μg/mL respectively after 72 h (Table 1). Positive controls were strongly active against *O. ochengi* with LC_50_ of 2.23 ± 1.96 μg/mL for ivermectin, 3.62 ± 1.88 μg/mL, for levamisole and 4.34 ± 0.71 μg/mL for albendazole after 72 h incubation (Table 1). The various extracts of *L. lanceolata* showed anthelmintic activity; that confirms their use in the traditional treatment of filariae. The ethanolic and the MeOH-CH_2_Cl_2_ extracts of *L. lanceolata* have shown an anthelmintic activity similar to ivermectin, levamisole and albendazole after 48 h and 72 h post incubation (*P* < 0.05).

**Table 1:** LC50 of *L. lanceolata* crude extracts and positive control tested against *O. ochengi* and *C. elegans* wild type after 48 h and 72 h exposure. Data are mean ± SEM from three independent duplicate experiments

LC_50_ μg/mL after 72 h (after 48 h)									
	Ethanolic extract			Methanolic/methylene chloride extracts			Positive controls		
Worms	Leaves	Trunk barks	Root barks	Leaves	Trunk barks	Root barks	Ivermectin	Levamisole	Albendazole
*O. ochengi*	**9.76 ± 0.49** ^**ns**^ (11.68 ± 0.44^ns^)	**8.05 ± 1.15** ^**ns**^ (9.26 ± 1.67^ns^)	**6.39 ± 2.11** ^**ns**^ (7.69 ± 1.35^ns^)	**9.45 ± 0.37** ^**ns**^ (12.33 ± 1.01^ns^)	**7.95 ± 1.70** ^**ns**^ (10.77 ± 2.55^ns^)	**6.39 ± 2.11** ^**ns**^ (7.63 ± 1.29^ns^)	**2.23 ± 1.96** ^**ns**^ (5.27 ± 0.01 ^ns^)	**3.62 ± 1.88** ^**ns**^ (6.93 ± 0.032 ^ns^)	**4.34 ± 0.71** ^**ns**^ (8.001 ± 0.00 ^ns^)
*C. elegans*	**4650.00 ± 1.58** ^**^ (8210.00 ± 2.71^ns^)	**1200.00 ± 0.47** ^**ns**^ (2370.00 ± 0.66^*^)	**1890.00 ± 0.26** ^**^ (3030.00 ± 0.92^**^)	**3530.00 ± 0.78** ^***^ (5440.00 ± 1.45^***^)	**2030.00 ± 0.36** ^**^ (2070.00 ± 0.39^**^)	**1000.00 ± 0.33** ^ns^ (2640.00 ± 0.52^*^)	**2.17 ± 0.66** ^**^ (2.41 ± 0.33 ^ns^)	**4.12 ± 0.31** ^**^ (4.15 ± 0.68^ns^)	**4.26 ± 0.00** ^**^ (4.35 ± 0.57 ^ns^)

### Anthelmintic activity of ethanolic and methanolic/methylene chloride extracts of *L. lanceolata* against *C. elegans* WT and drug resistant strains

**Fig. 2:**
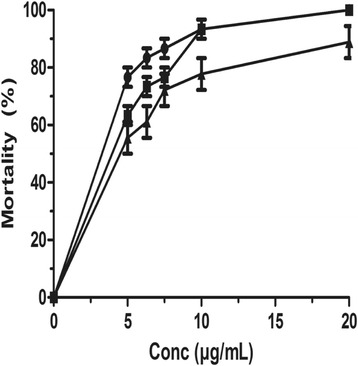
Activity against *C. elegans* with reference drugs 72 h post-exposure: (●) Ivermectin, (▲) Albendazole, (■) Levamisole. Data are mean ± SEM from three independent duplicate experiments

On the wild type of *C. elegans*, ethanolic and MeOH-CH_2_Cl_2_ extracts of leaves, trunk bark and root bark of *L. lanceolata* exhibited moderate activity. Worm mortality increased with concentrations (Fig. 2). The lowest concentrations required to inhibit 50% mortality (LC_50_) were 1890.00 ± 0.26 μg/mL, 1200.00 ± 0.47 μg/mL and 1000.00 ± 0.33 μg/mL, 2030 ± 0.36 μg/mL after 72 h respectively for root bark and trunk bark of *L. lanceolata* (Table 1).

The mortality as shown in Fig. 2 induced by ivermectin, levamisole and albendazole is time and concentration-dependent. These three drugs killed considerably the wild-type strain with the LC_50_ of 2.17 ± 0.66 μg/mL, 4.12 ± 0.31 μg/mL and 4.26 ± 0.00 μg/mL respectively after 72 h incubation (Table 1).

The ethanolic and the MeOH-CH_2_Cl_2_ extracts of the leaves of *L. lanceolata* showed activity with higher LC_50_ on *C. elegans* wild type strain compared to ivermectin, levamisole and albendazole after 72 h incubation (Table 1) (*P* < 0.01). Meanwhile, the trunk bark and the root bark showed the highest activity for ethanolic and MeOH-CH_2_Cl_2_ extracts respectively after 72 h incubation time.

**Fig. 3:**
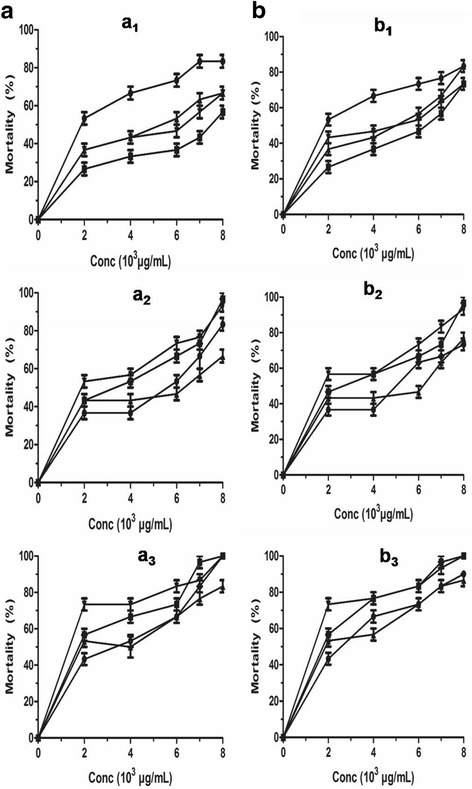
Effects of plant extracts against *C. elegans* wild type and drug resistant strains with (**a**) crude alcoholic and (**b**) MeOH-CH_2_Cl_2_ extracts from *L. lanceolata* 72 h post-exposure (●) VC722, (■) CB3474, (▲) CB211 and (▼)WT: (a_1_, b_1_) leaves; (a_2_, b_2_) trunk bark and (a_3_, b_3_) root bark. Data are mean ± SEM from three independent duplicate experiments

The anthelmintic activity of *L. lanceolata* was assessed in vitro against three resistant strains of the free-living nematode *C. elegans*, namely CB211 resistant to levamisole, CB3474 resistant to albendazole, VC722 resistant to ivermectine (Fig. 3). The anthelmintic activities of *L. lanceolata* leaves, trunk bark and root bark extracts were assessed in vitro on NGM-Agar. The in vitro activity of extracts on drug resistant mutants was concentration-dependent (Fig. 3a and b). *L. lanceolata* ethanolic leave extracts were strongly active against albendazole CB3474 and ivermectine VC722 resistant mutant strains with LC_50_ values of 1030 and 1170 μg/mL after 72 h respectively (Table 2 and Fig. 3a_1_).

**Table 2:** LC50 of *L. lanceolata* crude extracts and positive control tested against *C. elegans* wild type and ivermectin-, levamisole- and albendazole mutant resistant strains of the free living nematode *C. elegans* after 48 h and 72 h post-treatment. Data are mean ± SEM from three independent duplicate experiments

LC_50_ (μg/mL) after 72 h (after 48 h)									
	Ethanolic extract			Methanolic/methylene chloride extracts			Positive controls		
*C. elegans*	Leaves	Trunk barks	Root barks	Leaves	Trunk barks	Root barks	Ivermectin	Levamisole	Albendazole
**Wild type**	**4650.00 ± 1.58** ^**^ (8210.00 ± 2.71)^***^	**1200.00 ± 0.47** ^**ns**^ (2370.00 ± 0.66)^**^	**1890.00 ± 0.26** ^**^ (3030.00 ± 0.92)^***^	**3530.00 ± 0.78** ^***^ (5440.00 ± 1.45^***^)	**2030.00 ± 0.36** ^**^ (2070.00 ± 0.39^**^)	**1000.00 ± 0.33** ^ns^ (2640.00 ± 0.52^*^)	**2.17 ± 0.66** ^**^ (2.41 ± 0.33)^***^	**4.12 ± 0.31** ^**^ (4.15 ± 0.68)^**^	**4.26 ± 0.01** ^**^ (4.35 ± 0.57)^***^
**CB3474**	1030.00 ± 3.07*** (1039.00 ± 1.65) ^ns^	**2810.00 ± 0.10** ^**ns**^ (4267.00 ± 0.02) ^***^	**2030.00 ± 0.35** ^**ns**^ (2803 ± 0,16) ^ns^	**1470.00 ± 0.3** ^***^ (2830.00 ± 1.37 ^ns^)	**2620.00 ± 0.22** ^ns^ (3070.00 ± 0.34 ^***^)	**1860.00 ± 0.20** ^ns^ (2680.00 ± 0.25 ^ns^)	-	-	> 100
**CB211**	4220.00 ± 0.55** (7580.00 ± 2.38) ^ns^	**4720.00 ± 2.11** ^**ns**^ (1162.00 ± 3.49) ^***^	**2270.00 ± 0.66** ^**ns**^ (3063.00 ± 0.88) ^ns^	**3750.00 ± 0.32** ^ns^ (5510.00 ± 1.37^*^)	**3670.00 ± 0.75** ^ns^ (8790.00 ± 0.29^***^)	**2200.00 ± 0.36** ^ns^ (2940.00 ± 0.62 ^ns^)	-	> 100	-
**VC722**	1170.00 ± 0.60*** (5210.00 ± 2.61) ^ns^	**4120.00 ± 0.73** ^**ns**^ (7937.00 ± 1.65) ^ns^	**2850.00 ± 0.35** ^**ns**^ (5260.00 ± 5.10) ^ns^	**1820.00 ± 0.90** ^ns^ (5870.00 ± 1.47^ns^)	**3970.00 ± 0.55** ^ns^ (6920.00 ± 1.33^**^)	**2500.00 ± 0.37** ^ns^ (2620.00 ± 0.37 ^ns^)	> 100	-	-

In contrast, *L. lanceolata* trunk bark and root bark extracts display a very weak activity on the three drug resistant strains (Table 2 and Fig. 3a_2_, a_3_). Nevertheless, the effect of the ethanolic and MeOH-CH_2_Cl_2_ extracts of the root bark of *L. lanceolata* on the mutant strains CB3474, CB211 and VC722 was similar (ns) (2030, 2270, 2850 μg/mL and 1860, 2200, 2500 μg/mL respectively) after 72 h incubation (Fig. 3a_3_, b_3_). Statistical analysis of the effect of the leave extracts on the mutant strains of *C. elegans* presented in Fig. 3a_1_ and b_1_ revealed an important effect (*P* < 0.001) on VC722 and CB3474 (1170, 1030 and 1820 μg/mL) compared to the levamisole resistant strain CB211 (4220 and 3750 μg/mL *P* < 0.01) after 72 h (Table 2).

### Phytochemical dosages of ethanolic and methanolic/methylene chloride extracts of *L. lanceolata*

The quantification of phytochemical metabolites of the ethanolic and the MeOH-CH_2_Cl_2_ extracts were carried out to evaluate chemical families present in the plant extracts and which might be involved in the anthelmintic activity. The tannins, polyphenols, flavonoids and saponins were quantified; the results of these assays are shown in Table 3. In this table, it appears that polyphenol and tannin contents are the highest compared to flavonoids and saponins. Compared to ethanol, methanol/methylene chloride extracts more polyphenols and tannins (Table 3). Due to the high quantity of metabolites extracted in methanol/methylene chloride, this solvent was further used for fractionation.

**Table 3:** Phytochemical screenings of the ethanolic and MeOH-CH_2_Cl_2_ extract of leaves, trunk bark and root bark of *L. lanceolata*. The phytochemical screening revealed the presence of flavonoids, saponins, polyphenols and tannins in leaves, trunk bark and root bark of plants. Data are mean ± SEM from three independent duplicate experiments

	Ethanolic extract				Methanolic/methylene chloride extracts			
Parts used	(mg/g)							
	Polyphenols	Tannins	Flavonoids	Saponines	Polyphenols	Tannins	Flavonoids	Saponines
Leaves	414.07 ± 0.01	279.50 ± 0.01	8.76 ± 0.01	1.20 ± 0.05	1166.75 ± 0.01	558.00 ± 0,01	8.82 ± 0.02	1.20 ± 0.06
Trunk barks	394.52 ± 0.03	251.19 ± 0.01	25.34 ± 0.01	1.07 ± 0.05	2090.00 ± 0.04	1663.71 ± 0.09	58.00 ± 0.09	2.09 ± 0.06
Root barks	246.77 ± 0.04	166.40 ± 0.01	163.46 ± 0.01	2.05 ± 0.05	1880.00 ± 0.04	1333.00 ± 0.03	9.68 ± 0.03	5.12 ± 0.06

### Anthelmintic activity of *Lophira lanceolata* fractions against *Onchocerca ochengi* and *Caenorhabditis elegans*

During the screening of plant extract for anthelminthic activity, the crude alcoholic and MeOH-CH_2_Cl_2_ extracts of *L. lanceolata* leaves and root bark showed activity against the free-living nematode *C. elegans* and the cattle parasite *O. ochengi* (Tables 1 and 2). Leaves and root bark were fractionated and the 7 fractions of each were tested against *O. ochengi, C. elegans* WT and *C. elegans* drug resistant strains. Of the 7 fractions, fractions FHEAt, FEA and FEAM required higher concentrations to kill worms (Additional file 1: Table S1). The most active fractions were FH, FHEA, FEAMe and FM with LC_50_ between 3 to 5.70 μg/mL and 690 to 1850 μg/mL for *O. ochengi* and *C. elegans* WT respectively (Additional file 1: Table S1). These fractions therefore will be selected for analysis of their constituents.

### Assessment of acute toxicity of methanolic/methylene chloride extracts of *Lophira lanceolata*

In the study of acute toxicity test, oral administration of the ethanolic and the MeOH-CH_2_Cl_2_ extracts of leaves, barks of the trunk and root bark of *L. lanceolata* were assessed. In vivo studies revealed that no abnormal behaviour, no mortality during the treatment and observation periods was observed in animals treated at the doses 1500 mg/kg, 3000 mg/kg and 5000 mg/kg. Adverse reactions like increased motor activity, blinking eyes, tremors, convulsion, lacrimation, stimulation, muscle weakness, sedation, urination, salivation, lethargy, sleep, arching and rolling and coma up to a dose of 5000 mg/kg were not noticed within 14 days. These results confirm that, the doses tested were harmless for further in vivo investigations via gavage.

## Discussion

This study was undertaken to assess the anthelmintic efficacy of the crude extract of *L. lanceolata* against the bovine filarial nematode *O. ochengi* and the free-living nematode *C. elegans*. *O. ochengi* and *C. elegans* have widely been used to evaluate the efficacy of several anti-filarial agents [26–31]. This study investigates the nematotoxicity of the extracts and fractions of *L. lanceolata* against *O. ochengi*, *C. elegans* WT and three drug-resistant mutant strains (CB211, CB3474 and VC722). Results demonstrated that the parasite is significantly affected by the plant extracts than the free-living nematode. Results obtained after the exposure of *O. ochengi* to the leaves, the bark of the trunk and the root bark extracts of *L. lanceolata* reveal strong mortality.

Recent reports have revealed that *L. lanceolata* is used in traditional medicine against constipation, diarrhoea, dysentery, menstrual pain [12]. The pharmacological activity studies of this plant revealed that it possesses antipyretic activity, antimicrobial activities [13], antidiarrhoeal and anti-plasmodial effects [14]. Remarkably, *L. lanceolata* has never been tested against the bovine parasitic nematode *O. ochengi* and the free living nematode *C. elegans*. However, studies with other plants than *L. lanceolata* have been reported to show anthelmintic activities [17, 26–28, 31–34]. These studies, reporting anthelmintic activity of various plants, give an insight on the use of plants in folk medicine. Nevertheless, it has been shown that plants with anthelmintic activities contain phytochemicals such as polyphenols, tannins, flavonoids, saponins [28, 31, 33, 34] which may act synergistically to kill worms. The present work confirms this finding since polyphenols and tannins were the mainly metabolites extracted. The anthelmintic activity of *L. lanceolata* MeOH-CH_2_Cl_2_ extract was mainly related to polyphenols and tannins. These results confirm those of Prashant et al. [35]. These authors reported that polyphenols and tannins have anthelmintic activities. The presence of these metabolites can explain the high activity of this plant. The phytochemical study of MeOH-CH_2_Cl_2_ fractions revealed the presence of unevenly distributed bioactive elements (Additional file 1: Table S2). The tannin content of the methanol fraction of leave (FM) reflects its higher anthelmintic activity (Additional file 1: Table S1). Other fractions, although containing these chemical families, appeared to have no anthelmintic activity. This may be the result of their lack of solubility in RPMI and M9-Buffer. These results are similar to those of Mahmoudi et al. [36] who concluded that the solubility of phenolic compounds depends on their chemical nature in the plant, which varies from single to strongly polymerized compounds. The activity of *L. lanceolata* MeOH-CH_2_Cl_2_ extracts and fractions demonstrated on the filarial nematode *O. ochengi* and *C. elegans* might be due to the presence of these phytochemical products which might act synergistically. Due to the presence of tannins in *L. lanceolata*, mortality observed might be explained by the fact that tannins react directly with surface proteins of the worm. They cause physiological dysfunctions with regard of the mobility and the absorption of nutrients, leading to the death of worms as observed by Massamha et al. [37]. It has been demonstrated that tannins interfere with the production of energy in helminth parasites by decoupling the oxidative phosphorylation [38]. Another possible anthelmintic effect of tannins is that, they can bind to glycoproteins on the cuticle of the parasite and can indirectly cause death [39, 40]. These tannins activities might approve possible modes of action of *L. lanceolata* because the majority of chemical families in these plants are polyphenols and tannins. Mortality observed may also be the consequence of the presence of polyphenols. Polyphenols such as ellagic acid, gentisic acid and gallic acid have been shown to kill *O. ochengi* [34]. It has long been known and demonstrated in various studies that tannins and other polyphenolic compounds are protein coagulants which could result in a broad spectrum worm killing activity [41, 42]. Iqbal et al. [40] suggested that, condensed tannins may also bind to the cuticle of larvae which is rich in glycoprotein according to Thompson and Geary [39] and cause death [40]. On one hand, results of the fractions on *O. ochengi* are in the same range as those observed for some other fractions by Samje et al. [28] who tested the activity of *Craterispermum laurinum* and *Morinda lucida* on *O. ochengi* (LC_50_ ranked from 7.8 to 46.8 μg/mL). On the other hand, our results recorded lower range of values as compared to those found by Metuge et al. [27]. These authors tested secondary metabolites from *Cyperus articulates* on *O. ochengi* (LC_50_ of 15.7 μg/mL on males and 55.7 μg/mL on females). Some fractions are more active as compared to the crude extract while some others are less active. This may explain the synergistic effect of the crude extract. These results are similar to those observed by Rios and Recio [43] and Sarker et al. [44]. These authors concluded that the activity of an extract is probably due to the presence of synergy between a numbers of components, which when separated would become active in some fractions.

Results revealed a varying lethality of the three resistant *C. elegans* strains to the different parts of *L. lancealata*. CB211 is a knockout mutant of the genes *lev-9*. The gene *lev-9* is secreted in muscle cells and is responsible for locomotion and egg-laying. Compared to WT, mutant CB211 is slightly sensitive in the presence of leaves (Table 2). This result suggests that the mode of action of leave extracts of *L. lanceola* differs from that of levamisole. CB211 is resistant when incubated with the bark of the trunk or the root bark of *L. lanceolata* (Table 2), suggesting that these two parts may act similarly to levamisole. Levamisole belongs to the imidazothiazoles which are nicotinic receptor agonists [45, 46]. CB3474 is a knockout mutant of *ben-1*. This gene encodes β-tubulin that represents the binding site of albendazole, inhibiting the formation of microtubules [47, 48] and resulting in the paralysis of the worms [49]. Albendazole is one of the benzimidazole carbamates [45, 46]. Compared to wild type, mutants CB3474 is sensitive when incubated with leaves (Table 2). This result suggests that the mode of action of leave extracts of *L. lanceola* differs from that of albendazole. CB3474 is resistant when incubated with bark of the trunk or root bark of *L. lanceolata* (Table 2), suggesting that these two parts may act similarly to albendazole. Ivermectin is a drug classified amongst the macrocyclic lactones [46]. It is a GluCl receptor potentiator [50]. It specifically binds to GluCl channels and selectively paralyses the parasite by increasing muscle and nerve chloride-ion permeability thereby causing the death of worm [45]. VC722 is a single mutant in which the Glucl subunit *glc-2* has been knocked out. Glc-2 represents the binding site of ivermectin in pharyngeal muscle cells [8]. Compared to wild type, mutant VC722 is sensitive when incubated with leaves (Table 2). This result suggests that the mode of action of leave extracts of *L. lanceola* differs from that of ivermectin. VC722 is resistant when incubated with bark of the trunk or root bark of *L. lanceolata* (Table 2), suggesting that these two parts may act similarly to ivemectin. Results on the three mutants suggest that the efficacy on mutants is independent of genes transferring resistance to the strains and may be due to the chemical structures of molecules present in the different parts of the plant. Leaves of *L. lanceolata* thus appear to have a mode of action different to those of the commonly used anthelmintics, ivermectin, levamisole and albendazole.

Any test substance showing an LD_50_ of 5000 mg/kg after oral administration can be considered safe [51]. These results are similar to those observed by Ali et al. [13] evaluating the toxicity of *L. lanceolata* leaves in mice and having a mortality at the 4000 mg/kg dose. The result of the acute oral toxicity indicates that the plant extracts under study, when given orally, could be considered relatively safe.

## Conclusion

The present study assessed the ethanolic, the MeOH-CH_2_Cl_2_ extracts and fractions of leaves, trunk bark and root bark of *L. lanceolata* for in vitro anthelmintic activity by using the cattle parasite nematode *O. ochengi* and free-living nematode *C. elegans* as models. Our results showed the toxicity of *L. lanceolata* against *O. ochengi* and *C. elegans.* Therefore, these results are scientific basis which justify the use of *L. lanceolata* by traditional healers in the treatment of onchocerciasis and other worm infections. Moreover, *L. lanceolata* possesses significant anthelmintic potency without noticeable adverse effects in animal experiments. Further studies are required for HPLC or LC-MS analysis, to isolate and to characterize the bioactive constituents responsible of its anthelmintic activity.

## Additional file

Additional file 1:
**Table S1.** LC_50_ values of leaves and root barks of *L. lanceolata* fractions at 24 h post treatment against *O. ochengi* and *C. elegans* wild type and drug resistant strains. **Table S2.** Results of the quantification of fractions of leaves and root barks of *L. lanceolata (DOC 59 kb)*

## Abbreviations

ANOVA: One-way analysis of variance
FC: Folin-Ciocalteu
GluCls: glutamategated chloride channels
LF: Lymphatic Filariasis
MeOH-CH_2_Cl_2_: methanol/methylene chloride
MTT: Methyl-thiazol tetrazolium
nAChRs: nicotinic acetylcholine receptors
NGM: Nematode Growth Medium
NTDs: Neglected Tropical Diseases
OECD: Organization of Cooperation and Economic Development
