# Supplementary material for: Phytochemical analysis and in vitro anthelmintic activity of Lophira lanceolata (Ochnaceae) on the bovine parasite Onchocerca ochengi and on drug resistant strains of the free-living nematode Caenorhabditis elegans
Source: BMC Complement Altern Med. 2017 Aug 14;17:404. doi: 10.1186/s12906-017-1904-z (PMC5557511; doi:10.1186/s12906-017-1904-z)
Supplement: Additional file 1: — Table S1. LC50 values of leaves and root barks of L. lanceolata fractions at 24 h post treatment against O. ochengi and C. elegans wild type and drug resistant strains. Table S2. Results of the quantification of fractions of leaves and root barks of L. lanceolata (DOC 59 kb) [file 12906_2017_1904_MOESM1_ESM.doc]

**Supplementary Materials**

**Table S1:** LC_50_ values of leaves and root barks of *L. lanceolata* fractions at 24 h post treatment against *O. ochengi* and *C. elegans* wild type and drug resistant strains.

**Table S2:** Results of the quantification of fractions of leaves and root barks of *L. lanceolata*

**Table S1**

| **FH**   |  | **FHEA** | **FHEAt** | **FEA** | **FEAM** | **FEAMe** | **FM** |  | | --- | --- | --- | --- | --- | --- | --- | --- | | | | | | | | |
| --- | --- | --- | --- | --- | --- | --- | --- | --- | --- | --- | --- | --- | --- | --- | --- |
| **Worms** **Leaves of *Lophira lanceolata* (µg/mL)** | | | | | | | |
| *O. ochengi* (24 h) | 4.86 | 5.70 | 45.00 | 42.00 | 45.00 | 4.86 | 4.86 |
| WT | 1850.00 | 1850.00 | 4850.00 | 4550.00 | 3550.00 | 1450.00 | 1450.00 |
| VC722 | 2680.00 | 2680.00 | 2320.00 | 1660.00 | 1660.00 | 1190.00 | 1190.00 |
| CB3474 | 1640.00 | 3080.00 | 880.00 | 750.00 | 750.00 | 570.00 | 570.00 |
| CB211 | 3080.00 | 3080.00 | 880.00 | 750.00 | 750.00 | 570.00 | 570.00 |
| **Root barks of *Lophira lanceolata* (µg/mL)** | | | | | | | |
| *O. ochengi* (24 h) | 4.30 | 4.51 | 45.55 | 38.50 | 30.30 | 3.00 | 3.00 |
| WT | 1640.00 | 1640.00 | 1640.00 | 1640.00 | 1160.00 | 750.00 | 690.00 |
| VC722 | NT | 5480.00 | 5480.00 | 5480.00 | 3080.00 | 3080.00 | 1650.00 |
| CB3474 | NT | 1440.00 | 1440.00 | 520.00 | 520.00 | 520.00 | NT |
| CB211 | NT | 3640.00 | 3640.00 | 1240.00 | 1240.00 | 1240.00 | 920.00 |

FH= hexane (1:0 v/v), FHAE= hexane: acetate (8:2 v/v), FHAEt= hexane: acetate (6:4 v/v), FAE= acetate (1:0 v/v), FAEM= acetate: methanol (8:2 v/v),

FAEMe= acetate: methanol) (7:3 v/v) and FM= methanol (1:0 v/v), NT = Not Tested

**Table S2**

|  | **FH** | **FHEA** | **FEAM** | **FEAMe** | **FM** |
| --- | --- | --- | --- | --- | --- |
| **Metabolites** | **Leaves (mg/g)** | | | | |
| **Tannins** | 447.35 ± 0.003 | 631.26 ± 0.05 | 396.09 ± 0.02 | 818.66 ± 0.007 | 1052.34 ± 0.01 |
| **Polyphenols** | 836.84 ± 0.01 | 706.48 ± 0.01 | 1561.32 ± 0.00 | 1539.55 ± 0.01 | 1061.57 ± 0.01 |
| **Flavonoids** | 200.91 ± 0.01 | 189.80 ± 0.01 | 223.07 ± 0.007 | 220.19 ± 0.005 | 192.5 ± 0.006 |
| **Saponins** | 0.94 ± 0.01 | 0.94 ± 0.1 | 1.37 ± 0.0 | 1.80 ± 0.01 | 2.24 ± 0.01 |
| **Root barks (mg/g)** | | | | | |
| **Tannins** | NT | 68.80 ± 0.01 | 348.47 ± 0.01 | 266.73 ± 0.01 | 330.99 ± 0.01 |
| **Polyphenols** | NT | 80.20 ± 0.01 | 989.17 ± 0.01 | 671.72 ± 0.01 | 584.81 ± 0.01 |
| **Flavonoids** | NT | 181.34 ± 0.01 | 195.19 ± 0.01 | 225.76 ± 0.01 | NT |
| **Saponins** | NT | 0.86 ± 0.10 | 1.37 ± 0.01 | 1.37 ± 0.01 | 1.80 ± 0.01 |
